# Health beliefs and carer burden in first episode psychosis

**DOI:** 10.1186/1471-244X-14-171

**Published:** 2014-06-09

**Authors:** Maya Patel, Rajan Chawla, Carl R Krynicki, Philip Rankin, Rachel Upthegrove

**Affiliations:** 1 College of Medical and Dental Sciences, The University of Birmingham, Edgbaston, Birmingham B15 2TT, UK; 2 Birmingham Early Intervention Service, Birmingham and Solihull Mental Health Foundation Trust, Unit 1, B1, 50 Summer Hill Road, Birmingham B1 3RB, UK

**Keywords:** First episode psychosis, Carer burden, Locus of control, Illness appraisal, Ethnicity

## Abstract

**Background:**

Carer burden is high during First Episode Psychosis (FEP) and evidence suggests that this is a predictor of poor long-term outcome. However our understanding of factors associated with higher burden is poor. We propose that carers’ cultural backgrounds and health belief models will influence their perceived burden of care, over and above that explained by severity of illness.

**Methods:**

Patients with FEP and their primary Carers were recruited from the Early Intervention Service. Patients and Carers completed a range of validated measures, self-report ethnicity and demographic information together with the Multidimensional Health Locus of Control and Caregiver Burden Inventory.

**Results:**

Significant correlations were found between carer burden and health beliefs, which differed by ethnicity and gender. High physical burden was experienced by Black carers with an external locus of control; time restrictions and emotional burden correlated with an external locus of control in Asian carers. For White carers, external locus of control correlated with time dependence burden. In all ethnic groups female carers experienced more time dependency, physical and developmental burden. No significant correlations were found between patient measures of severity or duration of illness and carer burden.

**Conclusions:**

The type of burden experienced by carers differed between gender and ethnicity and was related to their health belief models. Thus the explanation and understanding of illness appears to be more salient than simply a patient’s severity of illness when considering the development of carer burden. Interventions to tackle high carer burden, and thus expressed emotion to improve outcome in patients, may need increasing focus here.

## Background

Families and other lay carers play a substantial role in the management of patients with mental illness [1]. In the UK alone there are thought to be around 1.5 million mental health carers [2]. Carers may undertake a wide range of practical tasks including supervising medication regimes, housework, shopping, and maintaining contact with mental health services. These duties place a significant burden on patients’ families, not least because of distress associated with disease symptomology, but also due to the social stigma associated with mental illness [3,4]. Carers must cope with the emotional and psychological consequences of looking after someone with a mental illness, including feelings of guilt, shame, worry and distress [5-7].

High levels of carer burden have been found to be present in various chronic illnesses such as dementia [8-10], bipolar disorder [11] and psychosis [3]. Carer burden is particularly high during first episode psychosis (FEP) [5], and there is evidence to suggest that this is a predictor of poor long-term outcome for the patient [12]. However our understanding of this association is poor. There may be a mediation of high carer burden through the influence of ‘expressed emotion’ (EE). EE is emotion expressed by close relatives towards a family member [13], and a high level of carer burden has been linked with a high level of EE in patients experiencing FEP [14]. High EE is present in over half of patient-carer relationships in FEP [15]. High EE is indicative of increased relapse rate in patients experiencing psychosis and schizophrenia [16], and is related to increased levels of anxiety and depression in both patients and carers [17].

Illness cognitions are common sense beliefs about illness [18] and provide a schema for coping with and understanding illness. Health beliefs influence health behaviours and have been shown to influence outcomes in a variety of illnesses in terms of accessing treatment and preventive interventions [19]. How an illness is understood or explained has the potential to affect the perceived burden experienced by the carer. Poor coping mechanisms in carers have been associated with increased anxiety, depression [20] and high EE [21]. Thus it is plausible that illness perceptions play a role in the experience of burden felt by carers. In addition a patient’s awareness and perspectives of his/her own illness is influenced by their perception of illness and treatment [22], and can profoundly affect care seeking behaviour and adherence to recommended interventions [23]. The beliefs and perspectives of their carer and the local culture will also have an influence upon this [24].

Several factors have been identified that influence the burden experienced by carers [25] including the relationship of the carer to the patient, the age of the carer [26], and the social support received by the carer [27]. The gender of the carer also plays an important role in the amount of burden experienced, with a greater level of burden being experienced by women [28,29]. Cultural differences seem to play a vital role in both patients’ and carers’ illness perceptions [30,31]. We have previously reported that differences exist in illness appraisals in Black and Minority Ethnic (BME) groups experiencing psychosis [32], with BME groups appraising their illness less negatively than other ethnic groups. Also Magliano et al. [33] found that patients experiencing FEP living in the Mediterranean were more likely to employ more spiritual methods of coping strategies when faced with caring for a FEP patient. Greater levels of emotional distress and burden have also been observed in carers of African descent [34], with more than one third scoring highly on the Carer Burden Inventory [35]. However, these results have not been supported by subsequent research [36]. These findings suggest that there are several factors that may influence the development of carer burden and EE, which are essential to identify in order to provide appropriate support to all types of carers of patients experiencing FEP. Health belief models can potentially affect the quality of the care provided by a carer, the carer’s health and the patient’s prognosis following FEP. Yet, the nature and impact of health beliefs on carer burden in individuals with FEP remains under-studied and to date there has been little research on this important subject.

This study will therefore address gaps in the knowledge base for predictors of carer burden, and aims to investigate factors relating to a higher level of carer burden during FEP. Specifically we propose that carers’ cultural backgrounds and health belief models will influence their perceived burden of care, over and above that explained by duration of untreated psychosis, level of illness, insight and co-morbid illness.

## Method

A cross-sectional study, using quantitative data collected from questionnaires and a semi-structured interview was conducted with patients in recovery after FEP, as well as their main carers. Participants were recruited from the South Birmingham Early Intervention Services, part of the Birmingham and Solihull Mental Health Foundation Trust. Patients within Birmingham EIS receive on average 3 years of intensive case management, relapse prevention, vocational support and psychological interventions as needed, following a first episode of psychosis. The majority of patients have a diagnosis of schizophreniform disorder.

All patients who met the following inclusion criteria were invited to participate; i) aged 18-35 years; ii) capacity to consent; iii) in recovery after experiencing the FEP. Exclusion criteria included; i) primary diagnosis of substance misuse or organic mental disorders, and ii) Non-English speaking patients or carers. Recovery was defined as a clinical improvement in positive symptoms sufficient to allow discharge from intensive or hospital based treatment with capacity to engage meaningfully with usual treatment in the community teams.

Carers were identified by the patient as the caregiver who is most available and provided the most support emotionally and/or financially. Carers had to have ‘reasonable contact’ with the patient (face-to-face contact at least twice a week). Ethical approval for the study was sought and received (West Midlands NHS REC 11/H1208/1).

### Materials

Patient Measures:

● Beck Anxiety Inventory [37].

● Insight Scale [38].

● Calgary Depression Scale for Schizophrenia (CDSS) [39].

● Positive and Negative Syndrome Scale (PANSS) [40].

● Duration of Untreated Psychosis (DUP); calculated using information taken from the PANSS interview and clinical notes as the interval between onset of psychosis and onset of treatment. ‘Onset of psychosis’ and ‘onset of treatment’ follow the standard definitions used by Larsen et al. [41].

Carer Measures:

● Multidimensional Health Locus of Control (MHLC) [42]; a scale for measuring locus of control, comprising internal locus (the belief that health outcomes are under the individual’s own control), ‘chance’ locus of control (the belief that health outcomes are determined by chance or fate) and ‘powerful others’ locus (external locus of control centred around the belief that health outcomes are determined by the actions of other influential people such as clinicians or family members). The ‘doctors’ and ‘other people’ subscales are related to ‘powerful others’ (and therefore external locus of control) and measures the belief that patients rely completely on the help of others (such as doctors) to deal with their illness [43,44]. This was scored using a Likert scale, which was rated from one (strongly disagree) to six (strongly agree). This is a reliable, valid and well used method of testing locus of control [44] and has been used on a psychiatric population [45].

● Caregiver Burden Inventory (CBI) [46]. Used to measure both objective burden (i.e. disruption to the carer’s life as a result of caring for the ill person) and subjective burden (i.e. the psychological and emotional consequences of caring), with more weight placed on subjective burden. Carer’s responses for the 24 items are rated on a Likert scale from 0 (not at all descriptive) to four (very descriptive). The CBI has been shown to be a valid measure of carer burden in carers of patients experiencing FEP [47]. It measures five factors of burden; (i) ‘time dependence burden’, the carer’s burden as a result of time restrictions placed on the carer; (ii) ‘developmental burden’, the carer’s feelings of holding off their development in relation to their peers; (iii) ‘physical burden’, the carer’s feelings of chronic fatigue and damage to physical health; (iv) ‘social burden’, the carer’s feelings of role conflict and how they feel neglected by others; (v) ‘emotional burden’, the carer’s negative feelings towards their care receivers often, which can result in feelings of guilt and shame [47].

### Data analysis

Data were analysed using Statistical Package for the Social Sciences (SPSS v.21). No significant violations of statistical normality were noted, therefore parametric tests were applied to examine the associations between carer burden with patient and carer scores on standardised measures detailed above. Data were treated as interval and the analyses were one-tailed unless otherwise stated, with the Alpha level set at .05. A multiple stepwise linear regression analysis was built to describe the relationship between the measures used in the prediction of carer burden, including ethnicity, DUP, PANSS, BAI and MHLC scores.

## Results

### Patient and carer descriptive statistics and preliminary analysis

A total of 145 patients met inclusion criteria and were approached for the study. Of these, 41 had no identified carer, and 43 declined to participate as they did not want to consent to any research studies. Thus the total number of participants was 61. Of these, 44 (72%) were male and 17 (28%) were female, 49 (80%) single, 10 (16%) were married and 2 (3%) were described as ‘other’. The majority lived with their main carer (89%). In terms of ethnicity 35 (57%) were described as ‘White’, 17 (28%) as ‘Asian’ and 9 (15%) as ‘Black’. The mean age was 24.9 years (SD = 5.57, range 17-37). 31 patients (50%) had a history of any substance or alcohol abuse. Table 1 shows the mean scores for patient measures. There were no significant differences in clinical measures for patients by broad ethnic group, with the exception that Black patients had a longer DUP (Mann-Whitney-U, p < 0.05).

**Table 1:** Mean score (s.d) for patient measures by ethnic groups

**Patient measures**	**White**	**Asian**	**Black**	**Total**
CDSS	4.97 (4.45)	4.41 (3.44)	3.00 (5.12)	4.52 (4.29)
IS total	8.22 (1.90	6.52 (2.26)	7.44 (2.75)	7.63 (2.23)
Awareness of symptoms	2.97 (1.04)	2.70 (1.10)	2.77 (1.39)	2.86 (1.10)
Awareness of illness	2.68 (1.18)	1.47 (1.32)	2.11 (1.36)	2.26 (1.34)
Need for treatment	2.57 (0.67)	2.35 (0.82)	2.55 (0.58)	2.50 (0.70)
BAI	11.91 (7.96)	8.64 (10.50)	6.55 (7.53)	10.82 (8.80)
DUP* (days)	60 (141)	62 (202)	112 (229)**	79.0 (334)
PANSS positive	10.81 (3.71)	11.16 (4.44)	10.28 (2.56)	10.82 (4.59)
PANSS negative	11.21 (4.30)	15.00 (8.32)	8.57 (1.39)	11.74 (6.21)
General	25.43 (8.53)	27.16 (9.10)	20.14 (2.11)	25.11 (8.25)

*DUP Median reported in place of mean due to non-normal distribution of data.

**significant difference in DUP Median score on Mann-Whitney U p <0.05.

The total number of carers was also 61. Of these, 46 (75%) were female and 15 (25%) were male, mean age was 46 years (SD = 12.52, range 17-77) and carer ethnicity profile closely matched that of the patients. 31 (54%) were White, 17 (28%) were Asian and 9 (15%) were Black (4 refused to state their ethnic origin). Relationship of carers to participants included; 36 (61%) mothers; 9 (15%) fathers; 8 (13%) spouse/partner; and 6 (10%) siblings. The median number of hours spent caring per week was 28.

Carers generally had a high level of burden, mean CBI score of 30 (SD = 2.35) and highest scores in ‘time dependency’ (11.71) and ‘developmental’ (7.59) burden subscales. Table 2 shows mean, range and standard deviations for carer measures. There were no differences in mean CBI and MHLC total or sub scores by ethnic group.

**Table 2:** Mean score (s.d.) for carer measures by ethnic group

	**White**	**Asian**	**Black**	**Total**
CBI Total	28.69 (19.00)	33.19 (25.86)	26.69 (25.74)	30.00 (22.35)
Social	3.36 (2.91)	2.52 (2.93)	3.33 (4.84)	2.69 (3.21)
Emotional	2.13 (3.18)	2.64 (3.62)	0.66 (1.00)	1.94 (3.07)
Physical	5.62 (4.65)	6.25 (4.78)	5.13 (6.85)	5.67 (5.08)
Time dependency	10.26 (10.40)	13.00 (14.60)	11.22 (11.78)	11.71 (12.24)
Developmental	7.30 (4.92)	8.76 (6.70)	6.33 (5.54)	7.59 (5.53)
MHLC Total	62.86 (12.94)	65.26 (9.87)	63.55 (6.96)	63.83 (11.41)
Chance	19.89 (6.17)	18.60 (6.17)	19.44 (5.57)	19.79 (5.33)
Powerful others	19.31 (6.14)	21.93 (4.07)	20.66 (5.72)	20.04 (5.67)
Internal	23.65 (5.56)	24.73 (3.78)	23.44 (7.46)	24.02 (5.33)
Doctors	11.27 (4.04)	12.60 (3.11)	12.33 (4.06)	11.62 (3.82)
Other People	8.03 (3.23)	9.33 (2.52)	8.33 (3.50)	8.41 (3.08)

No significant differences on CBI or MHLC between groups.

### Correlations with carer burden

There were significant positive correlations between total CBI score and measures of the MHLC. Subscale analysis revealed correlations between MHLC ‘other people’ and CBI ‘developmental’, *r* = .299, *p* = .025; MHLC ‘other people’ and CBI ‘emotional’, *r* = .274, *p* = .041; MHLC ‘powerful others’ and CBI ‘emotional’, *r* = .276, *p* = .040; MHLC ‘chance’ and CBI ‘time’, *r* = .390, *p* = .033; MHLC ‘chance’ and CBI ‘physical’, *r* = .289, *p* = .031. Table 3 shows correlations between CBI and MHLC.

**Table 3:** Correlation between the CBI and MHLC

	**CBI Time**	**CBI Developmental**	**CBI Physical**	**CBI Emotional**	**CBI Social**	**CBI Total**
MHLC Other people	.040	.299*	.263	.274*	.127	.212
MHLC Doctors	.229	.085	.156	.188	.122	.226
MHLC Powerful others	.176	.221	.249	.276*	.145	.268*
MHLC Chance	.390**	.158	.289*	.036	.188	.343**
MHLC Internal	.203	-.034	-.131	.132	.036	.098

*denotes significance p < .05. ** denotes significance p < .01.

When looking at the influence of gender, there was a significant positive correlation between CBI ‘physical’ and MHLC ‘chance’; *r* = .546, *p* = .043 for males. For females, CBI ‘physical’ correlated with MHLC ‘powerful others’; *r* = .328, *p* = .034, and MHLC ‘other people’; *r* = .306, *p* = .049. Also for females, CBI ‘developmental’ burden correlated with MHLC ‘other people’; *r* = .349, *p* = .023 and CBI ‘time dependency’ correlated with MHLC ‘chance’; *r* = .478, *p* = .001.

Carer burden and MHLC associations were different by ethnic group. Subscale analysis revealed CBI ‘time dependence’ and MHLC ‘chance’ were significantly correlated for White carers; *r* = .391, *p* = .036. For Black carers, a significant positive correlation was found between CBI ‘physical’ and MHLC ‘other people’; *r* = .688, *p* = .041. For Asian carers, significant correlations were found between CBI ‘time dependence’ and MHLC ‘powerful others’; *r* = .514, *p* = .05, and CBI ‘emotional’ and MHLC ‘other people’; *r* = .528, *p* = .043.

No significant correlations in carer burden were found between total scores of patient factors and carer burden; anxiety (BAI and CBI, *r* = 0.137), duration of untreated psychosis (DUP and CBI, *r* = -0.131), severity of illness (PANSS total and CBI, *r* = -0.053) and insight scores (IS total and CBI, *r* =0.012).

### Regression analysis

In order to determine how strongly different factors may contribute to high carer burden, ethnicity, PANSS positive and negative scores, DUP, BAI, and MHLC subscale score variables were entered in to a stepwise linear regression model to predict the severity of CBI total score. Preliminary analyses were conducted to ensure no violation of normality or multicollinearity (*r* = 0.12-0.62). The final multivariate model revealed that only carer chance was a significant predictor of CBI total score, and explained 32% (*R*^*2*^ = 0.32) of the variance in CBI score, *F* change 5.48 (1-46), *p* = 0.02. No other variables were significant in the model.

## Discussion

The aim of this research was to investigate factors associated with high carer burden during FEP. Specifically we proposed that carers’ cultural backgrounds and health belief models will influence their perceived burden of care, over and above that explained by duration of untreated psychosis, level of illness, insight and co-morbid illness. In addition we explored whether gender may impact on perceived burden in carers during FEP. The results revealed that, whist there were no broad differences in burden by ethnic group, health beliefs were related to carer burden particularly for carers of Asian descent. In the regression model, belief that illness is caused by ‘chance’, not being within one’s own control, was significant in the prediction of overall carer burden. The belief that illness is due to chance is indicative of a lack of control either by the carer or patient, therefore suggesting that perceived feelings of helplessness may impact upon the burden that is perceived by the carer. This interesting finding supports the hypothesis that illness perceptions can influence the perceived burden experienced by the carer. Consistent with previous reports [48,49], this study found that the beliefs and illness perceptions of the carer influences the burden they experience, with an external locus of control (‘chance’) being associated with higher levels of psychological distress, across all ethnic groups examined. As poor coping mechanisms in carers have been associated with increased anxiety and depression [20], and those with an external locus of control have associated higher stress levels [50], this may explain why those with an external locus of control perceived more burden.

Univariate analysis revealed other significant differences in experience of burden. Carers scoring highly on the ‘powerful others’ loci of control (and therefore having an external locus of control) experienced more burden by restrictions placed on time and the emotional burden associated with caring for these individuals. This suggests that there are different types of burden experienced by the carer which are associated with the individuals’ health beliefs. Additionally, carers of Black descent with an external locus of control experienced higher ‘physical’ burden. ‘Physical’ burden includes feelings of chronic fatigue and damage to physical health [46]. This is in contrast to Ukpong [34] who found that carers of Black descent experience more ‘emotional’ burden in FEP. However, given that this research was undertaken in Nigeria, there may be cultural differences within the differing Black descent group. Our results do suggest, however that the level of burden and health beliefs are related, with different types of burden being perceived by different ethnic groups. The presence of cultural differences in illness appraisals supports research by Magliano et al. [33] who found cultural differences in coping mechanisms by carers in FEP.

The present study confirmed gender differences in carer burden and health beliefs models thus supporting Rudnick [29] and Weimand et al. [28]. Specifically, males with a ‘chance’ locus of control experienced more ‘physical’ burden. Women with an external locus of control (including ‘chance’, ‘powerful others’ and ‘other people’ dimensions) experienced higher levels of ‘time dependency burden’, ‘physical burden’ and ‘developmental burden’. ‘Developmental burden’ includes feelings of being developmentally misaligned compared to their peers, which causes anxiety and strain [46]. However, given that a disproportionate number of carers were women, only tentative conclusions can be drawn when considering gender differences and perceived burden. Interestingly, those with an external locus of control seemed to experience a high level of burden, supporting the assumption that an external locus of control correlates with higher levels of psychological distress [48,49]. This not only suggests that there are differences between males and females in carer burden and health belief models but it also suggests possible mechanisms for these differences.

The research also examined the level of carer burden in relation to patient measures of severity of illness; however, we found no significant correlations. The level of carer burden did not correlate with the duration of untreated psychosis, the patients’ anxiety, patients’ insight or severity of illness manifestation. The finding that the manifestation of the patients’ psychosis and the duration of untreated psychosis do not seem to contribute to the level of burden experienced by the carer is surprising, as duration of untreated psychosis has previously been linked with higher levels of expressed emotion [51]. Perhaps most surprising was the lack of negative correlation between patient insight and carer burden. It would seem to follow that as poor patient insight and awareness of their illness has been associated with non-compliance with medication [52] and disengagement with services [53], this would place extra strain on carers who usually take up the role of managing their illness [54]. However, this was not found in the present research. Our findings suggest complex relationships between health beliefs and burden rather than simply severity of illness.

### Methodological limitations

The number of participants provided adequate power to compute statistical analyses, and also reflected the socio-economic and ethnic communities in Birmingham. However, these numbers may not be sufficient to detect significant correlations when considering both ethnicity and gender in carer burden. In addition a high number of participants did not consent to the study, or had no contact with relatives, and it is possible these participants and more difficult or different relationships with their carers not captured in our study. There were gender differences in our sample, and the majority of carers were female which will reflect on the generalizability of our results. Therefore, any conclusions based on this limited sample must be made tentatively. Using terms such as ‘White’, ‘Asian’ and ‘Black’ reduces a variety of cultures, customs and beliefs, which may differ within themselves, and could have impacted upon the results. The research excluded participants who could not speak English. This was largely due to practical constraints but may have had an impact on our findings, especially when analysing differences between ethnicities. Excluding these groups may have clouded the true nature of the differences. It is surprising that severity of illness and comorbid factors were not significantly associated with carer burden. It is possible that patients were sampled during periods of recovery and this result would be different if the sample were taken during periods of more severe illness, particularly during the initial development of acute illness. However, PANSS positive scores ranged from 7 to 21, indicating that patients with significant illness were included.

## Conclusion

The results suggest that there are differences in the perceived level of burden experienced by carers during FEP, which differs between ethnic groups, gender and health beliefs. Across all ethnic groups, an external locus of control was found to be associated with increased level of perceived burden. Gender differences were also detected with ‘physical’, ‘developmental’ and ‘time dependency’ burden being higher in female carers with an external locus of control, but only ‘physical’ burden being high for male carers with an external locus of control. This study has also begun to address gaps in the knowledge of differences between ethnicity and perceived burden of care in FEP and has attempted to describe the nature of these differences. However, the study did not find any significant correlations between duration of untreated psychosis, patient illness perception, insight, anxiety or psychosis manifestation, and carer burden. This suggests that carer perception of illness and health belief models are more significant characteristics when predicting perceived burden. These findings have therapeutic implications for family-based interventions and methods to improve patient outcomes through assessment and intervention strategies, which explore perceived lack of control for families and carers. If such targeted support were to be offered, and tailored to the individual, there may be potential to reduce perceived carer burden. Combating negative illness perceptions and encouraging perceptions of control and autonomy adopted in both carers and patients may accelerate patients’ recovery and improve patients’ and carers’ quality of life. Indeed the findings from a recent study have confirmed the conclusion that providing extra support through the use of bibliotherapy has had a beneficial effect on carers of those experiencing FEP [55]. However, further research is required in this field; exploring the differences between ethnic groups with larger samples allowing for more in-depth study of culture, rather than just ethnicity.

## Competing interests

The authors declare that they have no competing interests.

## Authors’ contributions

RU, MP and RC contributed to the development of the study, research design, acquisition of the data and drafting of the manuscript. PR contributed to the acquisition of the data and drafting of the manuscript. CK participated in the analysis of the data and drafting of the manuscript. All authors read and approved the final manuscript.

## Pre-publication history

The pre-publication history for this paper can be accessed here:

http://www.biomedcentral.com/1471-244X/14/171/prepub
